# Identification of genetic factors influencing flavonoid biosynthesis through pooled transcriptome analysis in mungbean sprouts

**DOI:** 10.3389/fpls.2025.1540674

**Published:** 2025-03-12

**Authors:** Yeonghun Cho, Hakyung Kwon, Byeong Cheol Kim, Donghwan Shim, Jungmin Ha

**Affiliations:** ^1^ Department of Agriculture, Forestry and Bioresources and Research Institute of Agriculture and Life Sciences, Seoul National University, Seoul, Republic of Korea; ^2^ Crop Genomics Lab, Plant Genomics and Breeding Institute, Seoul National University, Seoul, Republic of Korea; ^3^ Department of Plant Science, Gangneung-Wonju National University, Gangneung, Republic of Korea; ^4^ Department of Biological Sciences, Chungnam National University, Daejeon, Republic of Korea

**Keywords:** mungbean sprout, ultra-high-performance liquid chromatography, secondary metabolite content, sample pooling, RNA-Seq, gene expression

## Abstract

**Introduction:**

Mungbean (*Vigna radiata L*.) is gaining increasing interest among legume crops because of its nutritional value. Various secondary metabolites that act as antioxidants and bioactive compounds are beneficial for human health. The secondary metabolite content in plants is easily influenced by environmental conditions, and this influence varies depending on the genotype.

**Materials and Methods:**

Here, we screened six genotypes with consistently high and low content of major secondary metabolites (gallic acid, chlorogenic acid, neo-chlorogenic acid, genistin, formononetin, catechin, syringic acid, and resveratrol) across environmental replicates. Transcriptome data obtained from the individual genotypes were pooled into two groups: high and low levels of secondary metabolites.

**Results and Discussion:**

Of the 200 differentially expressed genes identified using stringent criteria, 23 were annotated in the secondary metabolite pathway. By combining the results of the secondary metabolite and transcriptome data, we identified six key genes encoding four enzymes (*CCoAOMT1*; Caffeoyl-CoA O-methyltransferase, *CYP81E1*; 4'-methoxyisoflavone 2'-hydroxylase, *DFR*; dihydroflavonol-4-reductase, and *HCT*; shikimate O-hydroxycinnamoyltransferase) that commonly influence the content of secondary metabolites (catechin, chlorogenic acid, formononetin, and genistin) in mungbeans. Through regulatory network analysis, NAC042 and MYB74 transcription factors were identified. These transcription factors regulate the expression of four key genes in mungbean, *CCoAOMT1(Vradi02g00000724.1), CYP81E1(Vradi09g00002897.1), DFR(Vradi07g00001336.1)*, and *HCT(Vradi07g00000614.1)* leading to high flavonoid content.

**Conclusion:**

These results provide information on the common genetic factors involved in the production of secondary metabolites, which can improve the nutritional value of mungbeans and contribute to the development of elite mungbean cultivars.

## 1 Introduction

Mungbean (*Vigna radiata L.*) is a major legume crop with high economic and agricultural value. It ranks third among legumes in South Asia, after chickpeas and pigeon pea, with an average annual per capita consumption of up to 2 kg (Vijayalakshmi et al., 2003). Mungbeans are an important source of vegetable proteins and an important resource for the alternative meat industry (Dahiya et al., 2015; Pataczek et al., 2018). Mungbeans are grown extensively throughout Asia, particularly in countries such as Thailand, where they account for up to 83% of the total area under legumes (Vijayalakshmi et al., 2003). In general, mungbeans are well suited to intercropping systems, complement other crops, and provide key benefits, such as fixing nitrogen from the atmosphere, improving soil fertility, and reducing the need for chemical fertilizers. The crop also has a high market value, resulting in a high price per kilogram, making it an attractive option for farmers seeking both profitability and sustainability (Nair and Schreinemachers, 2020).

Recently, mungbeans have attracted increasing interest from customers owing to their high levels of secondary metabolites (Tang et al., 2014; Pataczek et al., 2018). One of the ways to consume mungbeans is to sprout them; the key benefit of this method is that germination increases the amount of antioxidants and other bioactive compounds (Ebert et al., 2017). Specifically, carotenoids, vitamin E, and various phenolic compounds in seeds increase after germination, which helps prevent chronic diseases in humans, such as inflammation and cancer (Kim et al., 2012; Nderitu et al., 2013; Basli et al., 2017; Teodor et al., 2020; Li et al., 2021).

To increase the levels of secondary metabolites and enhance the nutritional value of mungbeans, understanding the regulatory mechanisms of the biosynthetic pathways of target compounds and the underlying genetic factors remains crucial. Although mungbeans are rich in secondary metabolites compared to other legumes, only a few studies have been conducted on the biosynthetic pathways of secondary metabolites in mungbeans, with most studies on soybeans. Furthermore, previous studies attempting to increase the antioxidant components in mungbeans have focused on controlling stress factors (Lim et al., 2022a, 2022b), as most secondary metabolites in plants are produced as defense mechanisms against biotic and abiotic stresses (Hartmann, 2007; Zandalinas et al., 2017). However, the effectiveness of this approach varies among genotypes, because each genotype may have different sensitivities or tolerances to environmental factors (Kim et al., 2023a). Therefore, investigating the genetic factors that regulate the biosynthesis of secondary metabolites in mungbeans is necessary. To achieve this, eliminating environmental factors and varietal differences to identify the underlying genetic determinants remains imperative.

Gene pooling has been used to identify useful genetic factors for plant breeding and development, thereby minimizing genotype specificity (Cronn et al., 2012; Botnari et al., 2018). Genetic pooling methods have been used in various strategies to identify common genetic factors associated with target traits regardless of individual variation. In a previous study conducted on pigeon peas (*Cajanus cajan* L.), the QTL-seq approach was used to identify candidate genes for flowering and leaf shape (Singh et al., 2022). In sesame (*Sesamum indicum* L.), the same approach was used to identify loci that control lignan content (Kim et al., 2023b). Because of the unique characteristics of gene pooling, it can also be applied to non-model plants, including cultivars or wild varieties, and their pooled transcriptomes (Ward et al., 2012; Goyal et al., 2016).

In this study, we conducted transcriptome pooling of six mungbean species to identify the key genes that regulate the biosynthetic pathways of secondary metabolites. These findings provide information on the key genetic factors involved in the biosynthesis of secondary metabolites and contribute to improving mungbean varieties with enhanced nutritional value.

## 2 Materials and methods

### 2.1 Plant material and sample preparation

A total of 12 mungbean cultivars were used in the present study (**Supplementary Table S1**). Seeds were germinated by soaking in distilled water for 16 h at 37°C using an incubator (ISS-4075R, Jeiotech). The germinated seeds were moved to a sprout cultivator (ST001A, Sundotcom) and cultivated for three days at 28–30°C with a water spraying interval of 4 h and water spraying time of 2 min (Kim et al., 2021). After three days, the sprouts were harvested. Each sample of the 12 mungbean varieties consisted of 30 sprouts, which were divided into two groups of 15 sprouts each for secondary metabolite phenotyping and RNA extraction, respectively. The first group was dried at 70°C for 24 h in an oven (HQ-DO84, CORETECH) and then finely grounded for extraction with 70% ethanol (Supelco, cat. no. 1009831011) in the dark at room temperature. The other group was stored at -80°C for further study and RNA extraction.

### 2.2 Measurement of flavonoid content using UPLC

Ultra-high-performance liquid chromatography (UPLC) analysis was performed to measure the metabolite content (Nexera series equipped with MPM-40, SCL-40, SPD-M40, LC-40, SIL-40, and CTO-40 units from Shimadzu, Kyoto, Japan) with a photodiode array detector. Separation was conducted using a ZORBAX SB-C18 column (3.5 μm, 4.6 x 150 mm; Agilent, PN 863953-902, Santa Clara, USA). The sample injection volume was 2 µL and the column oven temperature was set at 40°C. The solvent for the mobile phase gradients was ultrapure water (Thermo Fisher, W5-4, Korea with 0.1% acetic acid solution [v/v; solvent A] and acetonitrile [solvent B]). The ratio of solvent A proceeded with 95% and solvent B flowed at 1 mL/min as follows: 0–10 min 95–90% A, 10–11 min 90–85% A, 11–15 min 85–80% A, 15–16 min 80–70% A, 16–25 min 70–65% A, 25–28 min 65–50% A, 28–32 min 50% A, 32.1 min solvent A was increased from 50 to 95%, and 32.1–40 min 95% A. The chromatograms of reference compounds, biochanin A (Chemfaces, CFN99734, Wuhan, China), catechin (Chemfaces, CFN99646), genistin (Chemfaces, CFN90250), caffeic acid (Chemfaces, CFN99646), daidzein (Chemfaces, CFN98774), daidzin (Chemfaces, CFN99101), formononetin (Chemfaces, CFN99962), gallic acid (Chemfaces, CFN99624), genistein (Chemfaces, CFN98681), glycitein (Chemfaces, CFN99106), glycitin (Chemfaces, CFN99105), isovitexin (Chemfaces, CFN98620), neochlorogenic acid (Chemfaces, CFN97472), p-coumaric acid (Chemfaces, CFN98794), resveratrol (Chemfaces, CFN98791), syringic acid (Chemfaces, CFN98884), t-ferulic acid (Chemfaces, CFN92394), vitexin (Chemfaces, CFN98601), chlorogenic acid (Chemfaces, CFN99116), coumestrol (Chemfaces, CFN96040), kaempferol (Chemfaces, CFN98838), myricetin (Chemfaces, CFN98877), and quercetin (Chemfaces, CFN99272) were extracted at wavelengths ranging from 270 to 330 nm (**Supplementary Table S2**). The analysis results were determined with three replicate.

### 2.3 RNA extraction and cDNA library construction

Total RNA was extracted from six cultivars of mungbean sprouts using a Ribospin Plant RNA extraction kit (GeneAll, Songpa-gu, South Korea) following the manufacturer’s protocol. cDNA libraries for RNA-seq were constructed using a TruSeq Staranede mRNA LT Sample Prep Kit (Illumina Inc., San Diego, CA, USA). Twelve libraries were constructed with two libraries from each of the six cultivars used for replication. The size and quality of libraries used for sequencing were determined using a 2100 BioAnalyzer (Agilent Technologies, Santa Clara, CA, USA). Sequencing runs were conducted in the paired-end mode using a Truseq SBS Kit on the Illumina NovaSeq 6000 platform. The RNA sequencing data were deposited in the NCBI SRA database (PRJNA1086206).

### 2.4 Identification and analysis of differentially expressed genes

The reference genome and gene annotation data for mapping were downloaded from the Seoul National University Crop Genomics Lab (http://plantgenomics.snu.ac.kr) (Ha et al., 2021). The reads were mapped to the mungbean reference genome using HiSat2 (Graph-based genome alignment and genotyping with HISAT2 and HISAT-genotype | Nature Biotechnology, n.d). Counts per million of mapped read values were calculated using FeatureCounts (Liao et al., 2014). The raw read counts were normalized using the trimmed mean of M-values (TMM) method, and differentially expressed genes (DEGs) were identified using edgeR (Robinson et al., 2010). DEGs were defined as genes with an absolute log2foldchange (|log2FC|) ≥ 1 between the two groups.

### 2.5 Functional annotation of DEGs and iRegNet regulatory co-expression network analysis

Gene ontology (GO) (Gene Ontology Consortium, 2004) enrichment analysis was performed using Clusterprofiler (Yu et al., 2012). Kyoto Encyclopedia of Genes and Genomes ontology pathway enrichment analysis was conducted using ClusterProfiler. iRegNet regulatory co-expression network analysis was conducted with homologous genes from *Arabidopsis thaliana* detected using BLAST.

### 2.6 Validation of DEGs using real-time quantitative reverse transcription-PCR

Real-time quantitative reverse transcription PCR (qRT-PCR) was performed using a PrimeScript RT Reagent Kit with gDNA Eraser (TaKaRa Bio Inc., San Jose, CA, USA) according to the manufacturer’s protocols. Forty cycles of PCR amplification were performed using gene-specific primers. The relative gene expression levels were calculated using the $2^{-\Delta \Delta CT}\;$ method. The housekeeping gene, eukaryotic initiation factor 5A (*EIF5A*, Vradi07g21320), was used as a normalization control.

## 3 Results

### 3.1 Contents of secondary metabolites in mungbean sprouts

**Figure 1:**
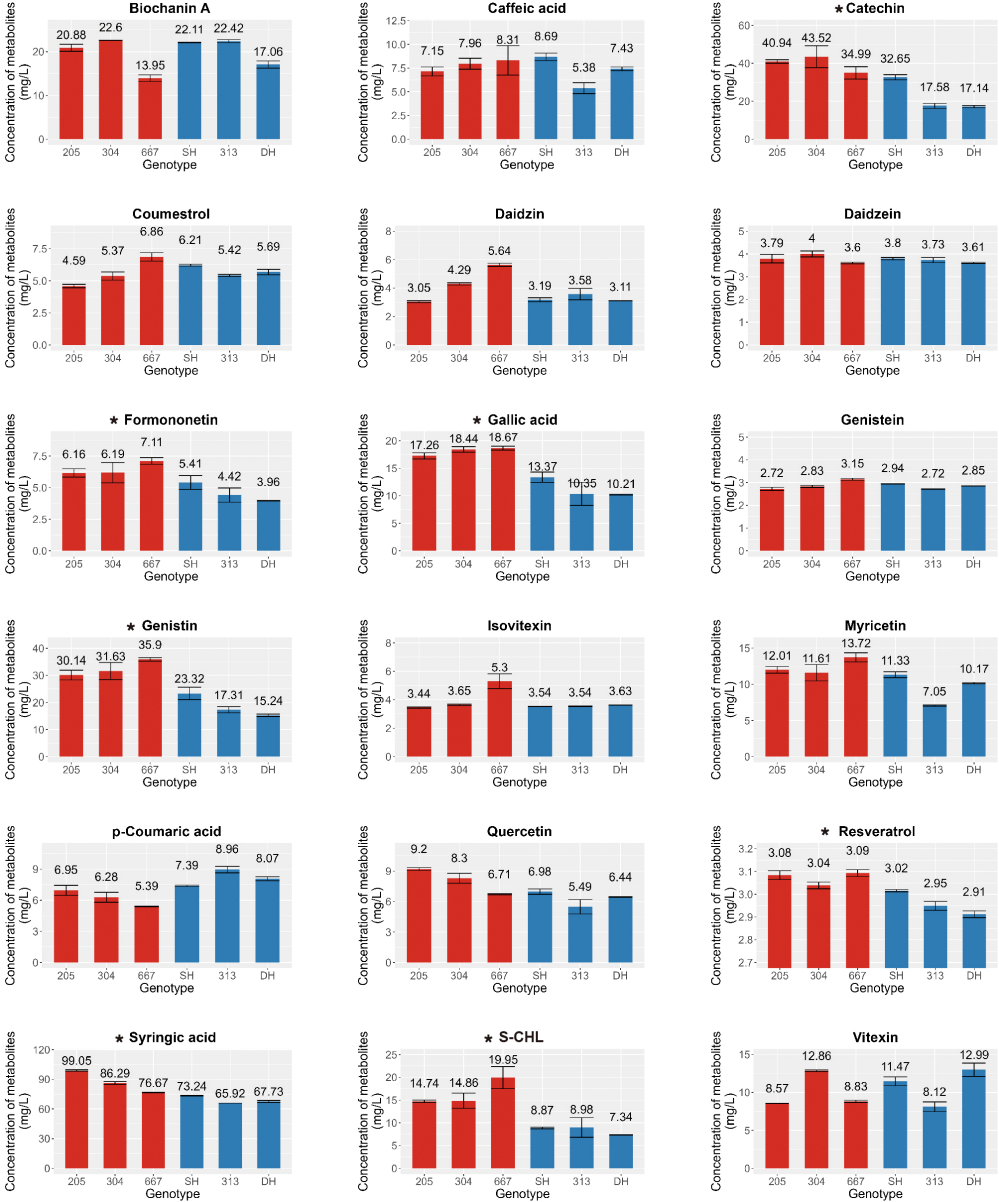
Contents of Secondary metabolites in mungbean sprouts of six genotypes. X-axis indicate mungbean sprouts genotype (205, 304, 667, SH, 313, and DH). Y-axes indicate the concentration of secondary metabolites (Catechin, Syringic acid, Genistin, Formononetin, Total Chlorogenic acid, Gallic acid, Resveratrol, Daidzein, Genistein, Vitexin, p-Coumaric acid, Caffeic acid, Daidzin, Isovitexin, Myricetin, Quercetin, Coumestrol, Biochanin A, and S-CHL, sum of chlorogenic acid and neo-chlorogenic acid.), determined with three replicates. The error bar indicates the standard deviation. The asterisk indicates metabolites that were statistically significant based on the ANOVA test (*p* < 0.05). Red and blue bars indicates high group and low group, respectively.

In a previous study on the metabolic profiling of 50 mungbean genotypes, 12 genotypes were selected based on the criteria of significant variations in the contents of 23 major secondary metabolites, and an expression analysis was conducted to identify key genes involved in the biosynthesis of secondary metabolites (Kim et al., 2023a). Building on these findings, we analyzed these 23 secondary metabolites again in the 12 genotypes to further investigate differences in metabolite accumulation between genotypes with consistently high and low secondary metabolite contents. Among the 23 commonly detected secondary metabolites in legumes, 19 were detected due to species-specific differences, comprising biochanin A, caffeic acid, catechin, coumestrol, daidzein, daidzin, formononetin, gallic acid, genistein, genistin, isovitexin, myricetin, p-coumaric acid, quercetin, resveratrol, syringic acid, sum of chlorogenic acid and neo-chlorogenic acid and vitexin; however, four metabolites, glycitein, clycitin, kaempferol, and t-ferulic acid, were not detected (**Supplementary Table S3**). To ensure robustness in selecting target metabolites for genetic analysis, we prioritized metabolites and genotypes at consistent levels across trials. As determined by one-way analysis of variance (ANOVA) at a significance level of 0.05, the contents of gallic acid, sum of chlorogenic acid and neo-chlorogenic acid, genistin, formononetin, catechin, syringic acid, and resveratrol were significantly higher in genotypes 205, 304, and 667 than in SH, 313, and DH, which was consistent with the results of our previous study (**Figure 1**). Other metabolites showed no significant differences between genotypes. Based on the consistently high and low levels of these secondary metabolites, genotypes 205, 304, and 667 were designated as the high group, whereas SH, 313, and DH were classified as the low group. These groups were then selected for further transcriptome analysis to compare expression patterns and investigate the key genetic factors involved in the biosynthetic pathways of these secondary metabolites.

### 3.2 Identification of DEGs between the high group and low group

To investigate the genetic factors underlying the differences in the secondary metabolite content, the total mRNA of the six mungbean genotypes, 205, 304, 667, SH, 313, and DH, were sequenced. In total, 80 Gb of reads (6.6 Gb per library on average) were obtained from the six mungbean genotypes (**Supplementary Table S4**). In general, 96.97% of the reads were properly aligned with the mungbean reference genome (Ha et al., 2021).

**Figure 2:**
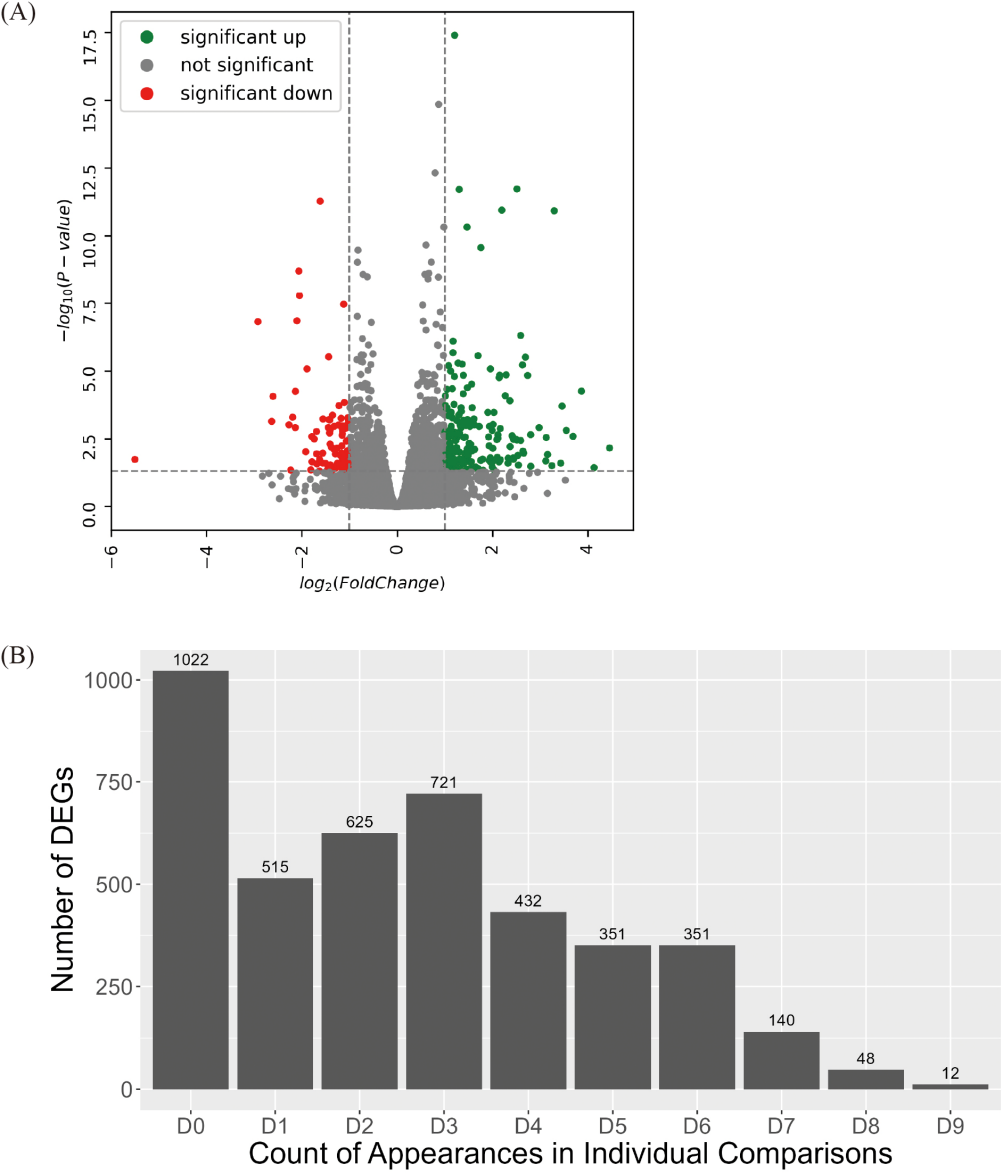
Identification of DEGs between high group and low group. **(A)** Volcano plot of DEGs identified between high group and low group. Colored dots represent DEG with value of 2foldchange. **(B)** Statistics of DEGs identified across each comparison between high group and low group. Among 4,217 DEGs between high group and low group, the number of DEGs cross-validated through individual comparison were identified. Every possible comparison between individuals in high group and low group were conducted. “D1” indicates the number of DEG identified between one genotype of high group and one genotype of low group. “D9” indicates the number of DEG appeared in every nine possible combinations between each genotype in high group and low group. “D0” indicates the number of DEGs unique to the high group and low group comparison.

To minimize genotype-specific variations, sequencing data from each genotype were pooled into high group and low group. Between the groups (high group vs. low group), 4,217 DEGs were identified, which comprised 1,838 upregulated and 2,379 downregulated genes (**Figure 2A**, **Supplementary Table S5**).

A group-wise comparison alone may not sufficiently capture consistent expression differences because DEG detection can be disproportionately influenced by a small number of genotype pairs exhibiting extreme variations. To minimize genotype-specific variations, a more stringent selection criterion was applied by analyzing DEGs through pairwise comparisons across all possible genotype combinations within the High and Low groups (**Figure 2B**). In total, nine pairwise comparisons were performed: Genotype 205 vs. Genotype SH, Genotype 205 vs. Genotype 313, Genotype 205 vs. Genotype DH, Genotype 304 vs. Genotype SH, Genotype 304 vs. Genotype 313,… Genotype 667 vs. Genotype DH. Each DEG was classified according to the number of pairwise comparisons in which it was detected. D1 denotes a gene that was identified in only one of the nine comparisons, and D9 denotes a gene that was consistently detected in all nine comparisons. After cross-validation, 200 DEGs were selected based on appearing in seven (D7), eight (D8), or nine (D9) pairs of comparisons. These genes with highly stable differential expression patterns were designated as true DEGs and subjected to further functional analysis.

### 3.3 Functional annotation of DEGs

**Figure 3:**
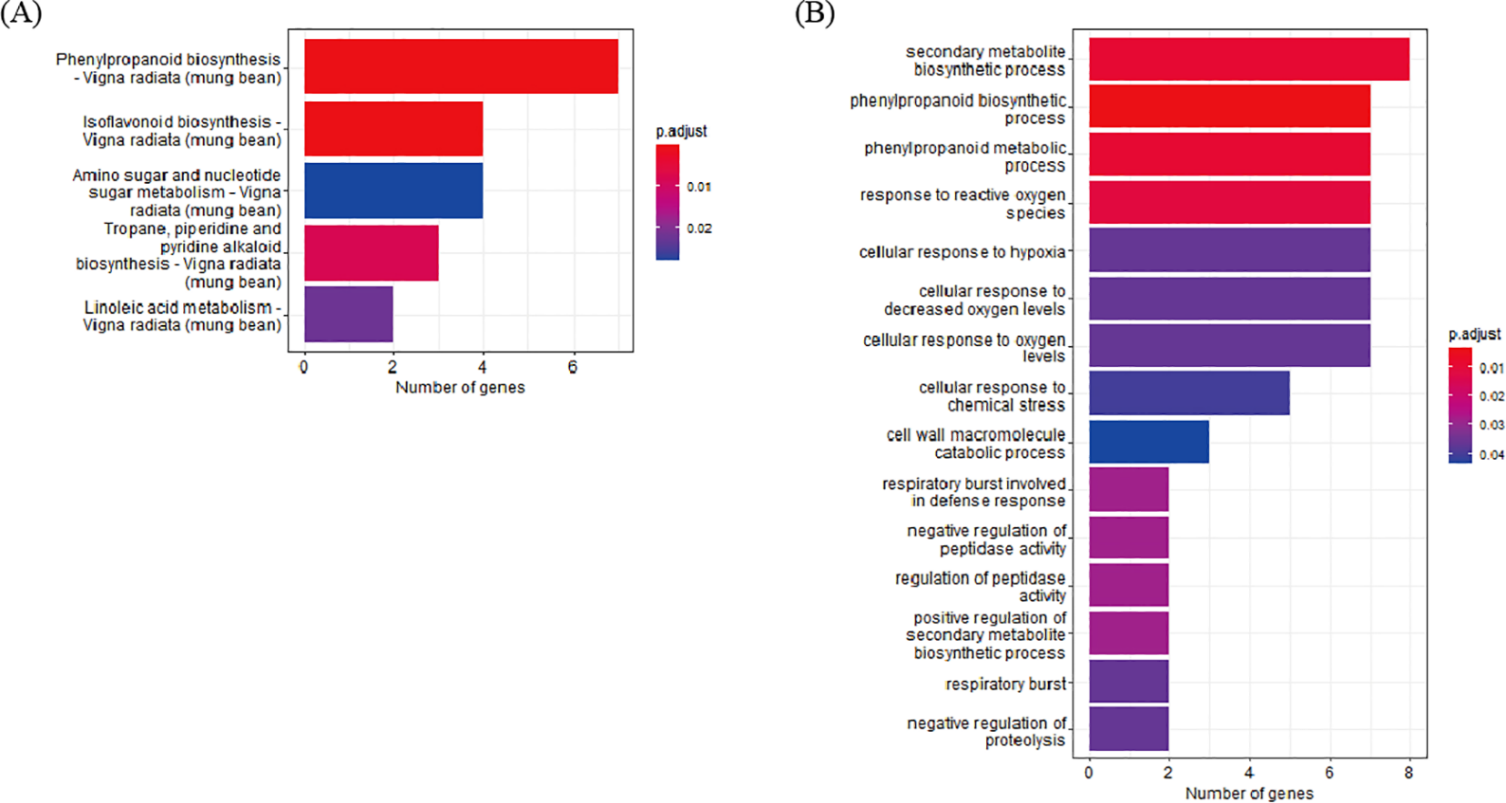
Enrichment analysis of DEG. **(A)** KEGG enrichment. **(B)** GO enrichment analysis of DEGs.

To determine the biological functions of DEGs identified through cross-validation, functional annotation of the 200 DEGs, which were identified at least seven pairs of combinations (D7, D8, and D9) was conducted using the Kyoto Encyclopedia of Genes and Genomes (KEGG) pathway and GO enrichment analyses. In the KEGG pathway analysis, 43 DEGs were annotated to five pathways and the top three pathways based on p-value harbor 14 genes; 4 genes in Isoflavonoid biosynthesis, 7 genes in Phenylpropanoid biosynthesis, and 3 genes in Tropane, piperidine, and pyridine alkaloid biosynthesis. In the GO analysis, 154 DEGs were annotated to 15 clusters. The top three clusters based on p-value include “phenylpropanoid biosynthetic process” (7 genes), “phenylpropanoid metabolic process” (7 genes), and “secondary metabolite biosynthetic process” (8 genes). Based on the two enrichment analyses, 23 DEGs were annotated as genes related to the biosynthetic pathways of secondary metabolites (**Figure 3A**, **Supplementary Table S6**).

### 3.4 Validation of gene expression levels using qRT-PCR

**Figure 4:**
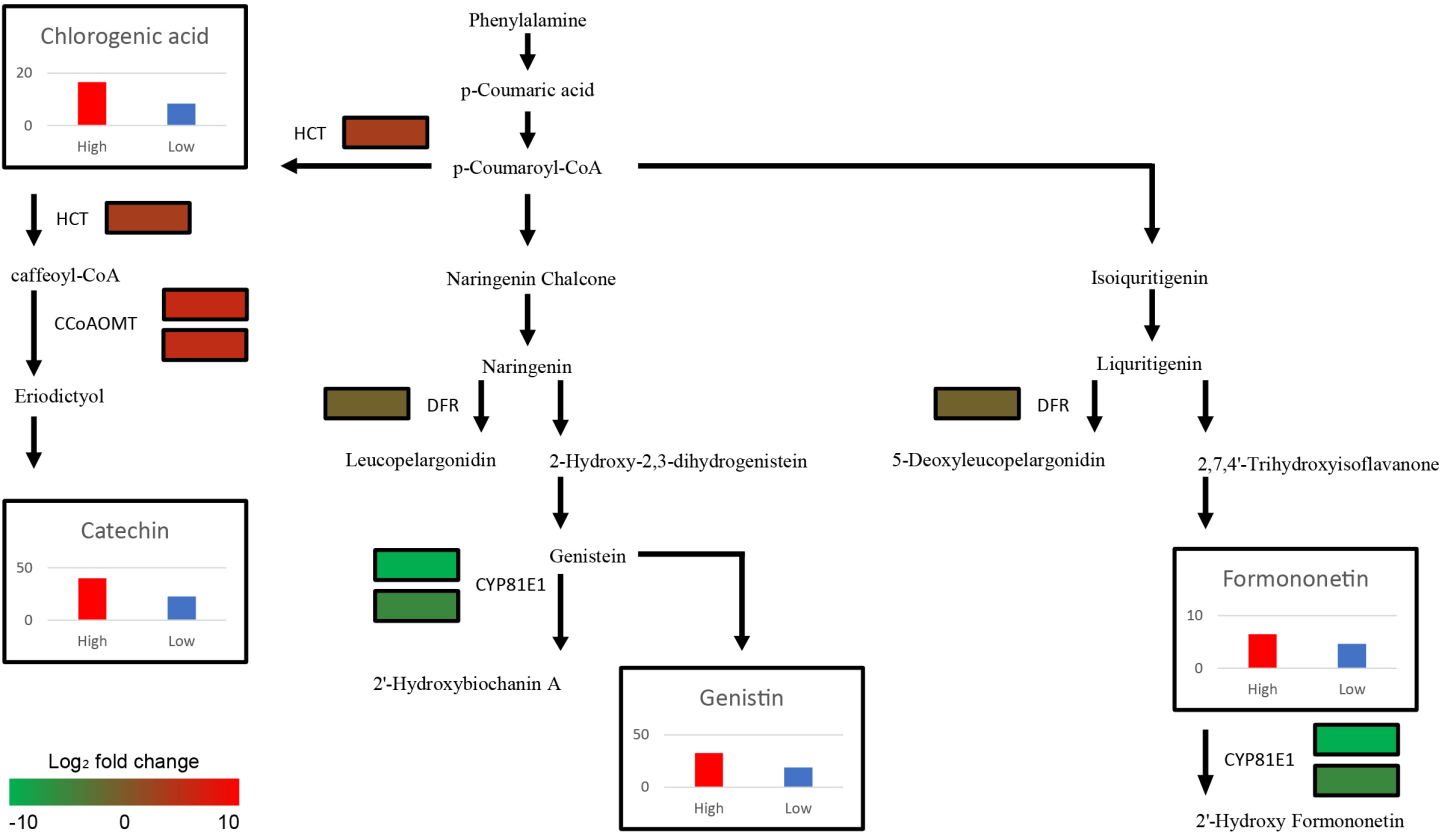
Schematic illustration of Secondary metabolite biosynthesis pathway with expression levels of 6 DEGs (CCoAOMT, CYP81E1, DFR, and HCT paralogs) and the average contents of 4 metabolites (Catechin, Chlorogenic acid, Formononetin, and Genistin) from each group. The color scale indicates fold change level of each DEGs between high group and low group. The bar plots show secondary metabolite content of High (Red bar) and Low group (Blue bar). The y-axis of four graphs represents concentration of metabolites (mg/L). CCoAOMT, caffeoyl-CoA O-methyltransferase; CYP81E1, 4’-methoxyisoflavone 2’-hydroxylase; DFR, dihydroflavonol-4-reductase; HCT, shikimate O-hydroxycinnamoyltransferase.

**Figure 5:**
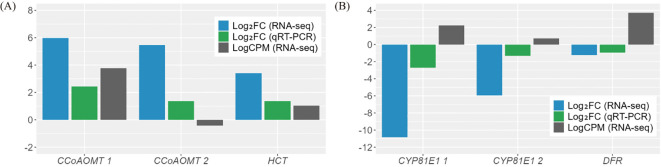
Validation of the DEGs through qRT-PCR analysis. **(A)** Up-regulated genes. **(B)** Down-regulated genes. The results of qRT-PCR were determined with three technical replicates. Log counts per million reads (LogCPM) values were used to identify key genes between paralogues for subsequent analysis. CCoAOMT, caffeoyl-CoA O-methyltransferase; CYP81E1, 4’-methoxyisoflavone 2’-hydroxylase; DFR, dihydroflavonol-4-reductase; HCT, shikimate O-hydroxycinnamoyltransferase.

Among the 23 DEGs, six were selected for qRT-PCR as key genes in the biosynthetic pathways that regulate the content of secondary metabolites (**Table 1**, **Supplementary Table S7**). These genes include caffeoyl-CoA O-methyltransferase (CCoAOMT: Vradi02g00000724.1, Vradi03g00001121.1), 4’-methoxyisoflavone 2’-hydroxylase (CYP81E1: Vradi09g00002897.1, Vradi02g00004162.1), dihydroflavonol-4-reductase (DFR: Vradi07g00001336.1), and shikimate O-hydroxycinnamoyltransferase (HCT: Vradi07g00000614.1), all of which are enzymes involved in the biosynthesis of chlorogenic acid, genistin, formononetin, catechin, syringic acid, and resveratrol (**Figure 4**). Consistent with the RNA-seq results, the expression levels of CCoAOMT and HCT were upregulated (**Figure 5A**), while CYP81E1 and DFR were downregulated in the high group than in the low group (**Figure 5B**, **Table 1**). These findings reinforce the reliability of our RNA-seq data, highlighting clear differential expression patterns that align with the distinct metabolic profiles observed in the high group and low group.

**Table 1:** RNA-seq result of six candidate genes used for qRT-PCR validation.

Gene name	Regulation	Gene ID	Log_2_ Fold Change	P-value	FDR	EC number	Description
CCoAOMT_1	Down	Vradi02g00000724.1	5.97	1.95.E-10	7.76.E-09	2.1.1.104	caffeoyl-CoA O-methyltransferase
CCoAOMT_2	Down	Vradi03g00001121.1	5.46	1.64.E-03	8.02.E-03	2.1.1.104	caffeoyl-CoA O-methyltransferase
CYP81E1_1	Up	Vradi09g00002897.1	-10.85	2.94.E-16	8.18.E-14	1.14.14.89	4’-methoxyisoflavone 2’-hydroxylase
CYP81E1_2	Up	Vradi02g00004162.1	-5.94	7.71.E-10	2.57.E-08	1.14.14.89	4’-methoxyisoflavone 2’-hydroxylase
DFR	Down	Vradi07g00001336.1	-1.24	2.11.E-05	1.91.E-04	1.1.1.219	dihydroflavonol-4-reductase
HCT	Down	Vradi07g00000614.1	3.41	9.60.E-08	1.72.E-06	2.3.1.133	shikimate O-hydroxycinnamoyltransferase

## 4 Discussion

Understanding the mechanisms by which secondary metabolites accumulate is necessary to enhance the nutritional value of mungbeans and develop elite cultivars. Previous studies have artificially induced stress conditions, including biotic and abiotic stresses, to increase secondary metabolite content (Singh et al., 2002). However, the response of different genotypes to these stress conditions varied with different response mechanisms against external factors (Kim et al., 2022) and certain genotypes showed consistently higher metabolite content with statistical significance compared to others under different conditions, suggesting the existence of genetic regulators that control genes and enzymes that influence the production of secondary metabolites.

Sample pooling has also been used in several genetic studies. For example, QTL-seq and genome-wide association studies use pooled DNA samples to identify candidate genes associated with target traits (Gyawali et al., 2019; Singh et al., 2022; Kim et al., 2023b). Although sample pooling offers optimized cost and statistical efficiency, previous research has revealed that it has the disadvantage of false detection caused by pooling bias (Wang and Paterson, 1994; Cutler and Jensen, 2010; Rajkumar et al., 2015). Despite this drawback, it remains a useful tool to identify common genetic variations, and researchers have tried various approaches to overcome false detections by overlapping identified DEGs from different methods (Ko and Van Raamsdonk, 2023). Therefore, in this study, we calculated DEGs using two combined approaches to minimize false detections. The first approach to calculating DEGs was RNA transcript pooling, grouping each member of the high group and low group as a replicate, resulting in 4,217 DEGs (**Figure 2A**). The second approach for calculating the DEGs was to compare each genotype of the high group with each genotype of the low group. With a total of nine possible combinations, DEGs detected from seven or more combinations were identified, and, in total, 200 DEGs were confirmed to be detected in HvsL DEGs (**Figure 2B**). Most of the 200 DEGs identified using our stringent criteria were annotated as being involved in secondary metabolite pathways according to KEGG (**Figure 3A**) and GO (**Figure 3B**) analyses, indicating that false detections were successfully removed from our DEGs.

Although the functions of major secondary metabolites in mungbeans are well known owing to their nutritional significance, the mechanisms of secondary metabolite biosynthesis in mungbeans remain unclear. Through functional annotation and the integration of metabolic and transcriptomic data, six genes encoding four key enzymes (CCoAOMT: Catechin, CYP81E1: Catechin and Chlorogenic acid, DFR: Genistin and Formononetin, and HCT: Genistin and Formononetin) were identified. *HCT* and *CCoAOMT*, which are involved in the biosynthesis of catechin and chlorogenic acid, showed higher expression levels in the high group genotypes, resulting in increased catechin and chlorogenic acid content in high group (**Figure 4**). These two metabolites, which are abundant in coffee and tea, offer many health benefits, including stable blood pressure and anti-cancer effects (Khalesi et al., 2014; Hayakawa et al., 2020). A previous study conducted on coffee (*Coffea canephora*) suggested that increased expression of *HCT* directly results in high levels of chlorogenic acid, which is consistent with the results of this study (Lepelley et al., 2007; Lallemand et al., 2012). In mungbean sprouts, chlorogenic acid content has been reported to be associated with the level of *HCT* expression under salinity stress (Kang, et al., 2022), which indicates that *HCT* is a key enzyme for the biosynthesis of chlorogenic acid in mungbeans. In *Arabidopsis*, eriodictyol accumulation has been reported to be caused by *CCoAOMT* (Wils et al., 2013), leading to increased catechin levels.

The expression levels of DFR and CYP81E1, which are involved in the isoflavonoid biosynthesis pathway, were significantly lower in high group with higher genistin and formononetin content than in low group (**Figure 4**) (Petit et al., 2007; Uchida et al., 2017). Naringenin and liquiritigenin, as intermediate precursors, are catalyzed by various flavonoids using these enzymes (Liu et al., 2021). Decreased expression of *DFR* and *CYP81E1* can activate an alternate biosynthetic pathway, resulting in the production of genistin and formononetin, which originate from naringenin and liquritigenin, respectively. The previous research findings, reporting that *CYP81E1* catalyzes the hydroxylation of the isoflavone formononetin, to yield 2′-hydroxyformononetin (Akashi et al., 1998), are consistent with our results, which demonstrate that the expression level of *CYP81E1* has a negative correlation with the formononetin content. The expression levels of *DFR* and *CYP81E1* in this study were lower in high group, which had higher genistin and formononetin content than low group (**Figure 4**). These results indicate that the downregulation of CYP81E1 and DFR may enhance the accumulation of genistin and formononetin in high group by redirecting biosynthetic pathways.

**Figure 6:**
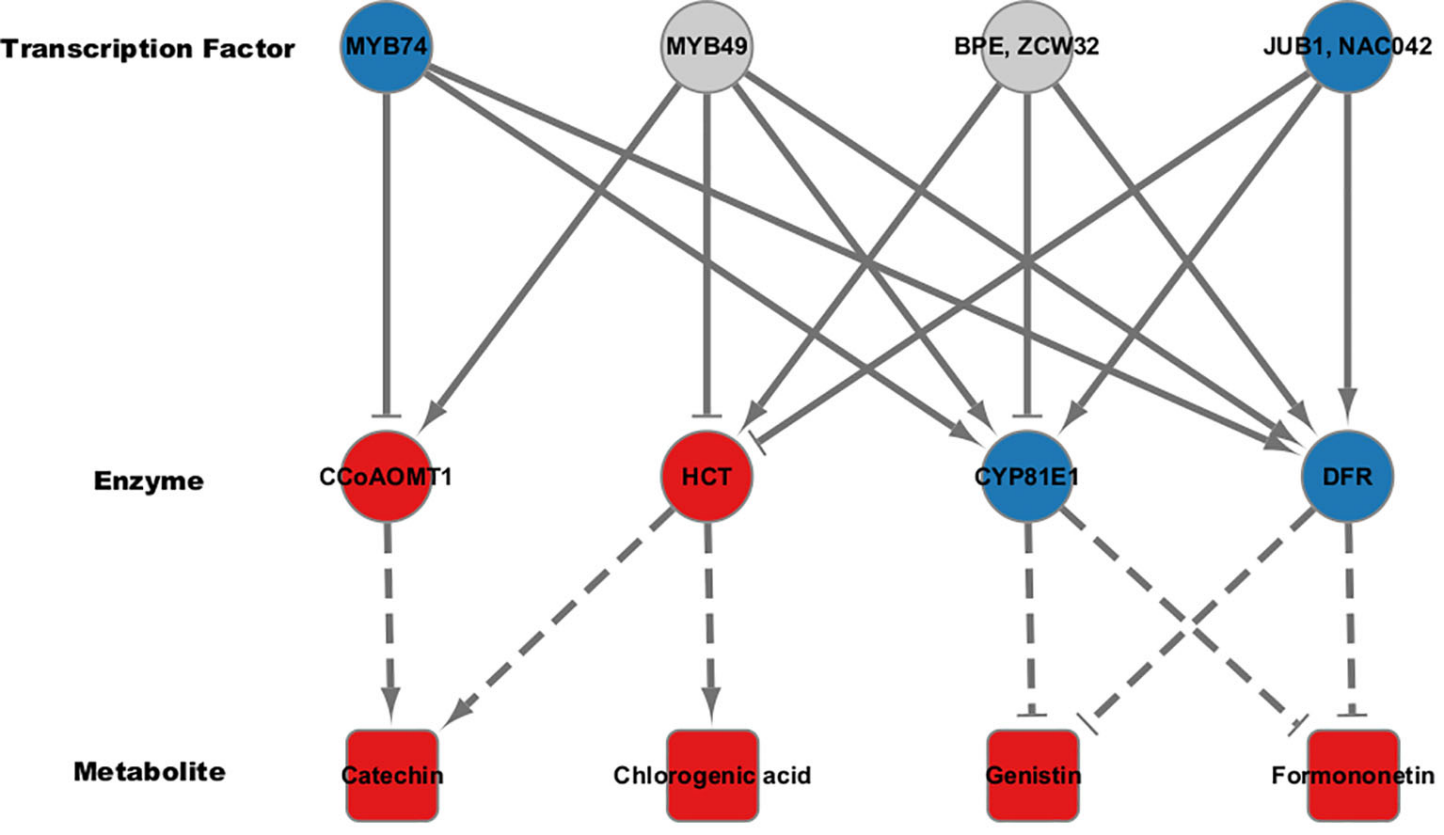
Regulatory co-expression network analysis for flavonoid biosynthetic pathway. Transcription factors affecting key enzymes in the flavonoid biosynthetic pathway were predicted using iRegNet. Arrows represent positive correlations, while T-shaped ends indicate negative correlations. Node colors reflect direction of regulation; Red for up-regulation and blue for down-regulation. Dotted lines suggest the association between the key enzymes and their corresponding secondary metabolites.

Our analysis revealed that the flavonoid content was generally elevated across different types of flavonoids in high group, with significant differences observed for chlorogenic acid, catechin, genistin, and formononetin (**Figure 1**). Additionally, despite the differences in the genetic backgrounds of the six mungbean genotypes, the expression levels of the six genes encoding the four key enzymes involved in the biosynthesis of these flavonoids were highly correlated. This suggests that these key enzymes are co-regulated by common genetic or transcriptional mechanisms, ultimately influencing flavonoid biosynthesis. To identify potential transcription factors upstream of these key enzymes, homologous *Arabidopsis* genes of these key enzymes were subjected to iRegNet analysis (Vradi02g00000724.1; AT4G34050, Vradi09g00002897.1; AT5G36220, Vradi07g00001336.1; AT5G42800, and Vradi07g00000614.1; AT2G19070) (**Figure 6**) (Shim et al., 2021). Four transcription factors regulate these enzymes: MYB49 (AT5G54230), MYB74 (AT4G05100), NAC042 (AT2G43000), and ZCW32 (AT1G59640). Among the mungbean homologs of these transcription factors, MYB74 (Vradi10g00000584.1) and NAC042 (Vradi06g00001115.1) showed significantly lower expression in high group compared to low group, with log2FC values of -3.68 and -4.3, respectively (**Table 2**). MYB74 and NAC042 are known to negatively regulate CCoAOMT1 and HCT, respectively, and positively regulate CYP81E1 and DFR. These findings suggest that the downregulation of MYB74 and NAC042 may have led to the upregulation of CCoAOMT1 and HCT and the downregulation of CYP81E1 and DFR, resulting in higher levels of catechin, chlorogenic acid, genistin, and formononetin in the high group.

**Table 2:** Fold changes of mungbean homologs of *Arabidopsis thaliana* transcription factors. “*A. thaliana*” refers to gene name from *Arabidopsis thaliana*, and “*V. radiata*” refers to gene names from *Vigna radiata*.

TF name	*A.thaliana*	*V.radiata*	Log_2_FC	p-value	Avg. RPKM	
					High group	Low group
MYB49	AT5G54230	Vradi04g00003889.1	N.D.	N.D.	16.512	24.66
MYB74	AT4G05100	Vradi10g00000584.1	-3.678	1.45E-05	749.90	1260.50
NAC042	AT2G43000	Vradi06g00001115.1	-4.295	2.427E-13	3488.04	7445.27
ZCW32	AT1G59640	Vradi07g00000781.1	0.500	0.004	30475.54	27157.72

TF, Transcription Factor; Avg, average; N.D., not detected in high group vs. low group DEGs.

While these findings provide insights into the transcriptional regulation of secondary metabolite biosynthesis, further studies are needed to validate the functional roles of key genetic factors. Such studies using CRISPR-based knockout, overexpression, and complementation test experiments will directly confirm their roles in flavonoid biosynthesis. Additionally, environmental factors influencing the expression of key genes and metabolite accumulation should be investigated to optimize growth conditions, develop elite cultivars, and enhance metabolite production in field. These approaches will provide insights into the regulatory mechanisms involved in secondary metabolism in mungbean.

In this study, we utilized metabolic data consistent with environmental conditions, along with DEGs identified using stringent criteria obtained from a pooled transcriptome. This approach aims to minimize environmental influences and eliminate variations due to genetic diversity among genotypes. The secondary metabolite content in mungbean sprouts was associated with the expression levels of the genes involved in the key biosynthetic pathways for each target metabolite. These results indicate that the accumulation or biosynthesis of secondary metabolites is regulated at the transcriptional level in mungbean sprouts. We identified the key genetic factors affecting the contents of major secondary metabolites. The findings of this study will contribute to the sustained and efficient production of antioxidant compounds in mungbean sprouts, thereby facilitating the development of high-value products with added benefits.

## Data Availability

The datasets presented in this study can be found in online repositories. The names of the repository/repositories and accession number(s) can be found in the article/**Supplementary Material**.
