# Supplementary material for: Identification of genetic factors influencing flavonoid biosynthesis through pooled transcriptome analysis in mungbean sprouts
Source: Front Plant Sci. 2025 Mar 12;16:1540674. doi: 10.3389/fpls.2025.1540674 (PMC11936997; doi:10.3389/fpls.2025.1540674)
Supplement: Supplementary file 1 [file Table1.docx]

Supplementary Material

**Supplementary table 1**. List of mungbean genotypes.

| Number | Accession name | Origin |
| --- | --- | --- |
| 205 | JP229216 | CHN |
| 216 | JP229099 | THA |
| 242 | JP103138-2 | PAK |
| 304 | Suwon No.2 | KOR |
| 313 | Gimje, Jeollabuk-do-1985-3835 | KOR |
| 331 | Yellowgram | UNK |
| 336 | Vo1301 | CHN |
| 362 | Namwon, Jeollabuk-do-1994-3237 | KOR |
| 667 | Tecer Hitam | IDN |
| DH | Dahyeon | KOR |
| SH | Sunhwa (VC1973A) | TWN |
| SM1409 | SM1409 | * |

^*^ Elite breeding lines

**Supplementary table** **2**. List of UPLC standard materials.

| No. | Compound | Cas No. | Chemical formula | Molecular weight | External ID | PubChem SID |
| --- | --- | --- | --- | --- | --- | --- |
| 1 | Biochanin A | 491-80-5 | C16H12O5 | 284.26 | C00814 | 4072 |
| 2 | caffeic acid | 331-39-5 | C9H8O4 | 180.15 | C01481 | 4652 |
| 3 | Catechin | 154-23-4 | C15H14O6 | 290.3 | C06562 | 8791 |
| 4 | Chlorogenic acid | 327-97-9 | C16H18O9 | 354.31 | C00852 | 4109 |
| 5 | Coumestrol | 479-13-0 | C15H8O5 | 268.2 | C10205 | 12391 |
| 6 | Daidzein | 486-66-8 | C15H10O4 | 254.2 | C10208 | 12394 |
| 7 | Daidzin | 552-66-9 | C21H20O9 | 416.38 | C10216 | 12402 |
| 8 | Formononetin | 485-72-3 | C16H12O4 | 268.27 | C00858 | 4115 |
| 9 | Gallic acid | 149-91-7 | C7H6O5 | 170.1 | C01424 | 4609 |
| 10 | Genistein | 446-72-0 | C15H10O5 | 270.2 | C06563 | 8792 |
| 11 | Genistin | 529-59-9 | C21H20O10 | 432.38 | C09126 | 11318 |
| 12 | Glycitein | 40957-83-3 | C16H12O5 | 284.26 | C14536 | 17395536 |
| 13 | Glycitin | 40246-10-4 | C22H22O10 | 446.41 | C16195 | 47205503 |
| 14 | Isovitexin | 38953-85-4 | C21H20O10 | 432.4 | C01714 | 4851 |
| 15 | Kaempferol | 520-18-3 | C15H10O6 | 286.2 | C05903 | 8191 |
| 16 | Myricetin | 529-44-2 | C15H10O8 | 318.2 | C10107 | 12293 |
| 17 | Neochlorogenic acid | 906-33-2 | C16H18O9 | 354.3 | C17147 | 96023599 |
| 18 | p-coumaric acid | 501-98-4 | C9H8O3 | 164.2 | C00811 | 4069 |
| 19 | Quercetin | 117-39-5 | C15H10O7 | 302.2 | C00389 | 3679 |
| 20 | Resveratrol | 501-36-0 | C14H12O3 | 228.2 | C03582 | 6374 |
| 21 | Syringic acid | 530-57-4 | C9H10O5 | 198.2 | C10833 | 13016 |
| 22 | t-ferulic acid | 537-98-4 | C10H10O4 | 194.2 | C01494 | 4664 |
| 23 | Vitexin | 3681-93-4 | C21H20O10 | 432.4 | C01460 | 4637 |

**Supplementary table 3**. Contents of secondary metabolites in 12 mungbean genotypes. (mg/L)

| **Genotype** | **Gallic acid** | **T-Chl** | **Catechin** | **Caffeic acid** | **Syringic acid** | **p-Coumaric acid** | **Daidzin** | **Vitexin** | **Isovitexin** |
| --- | --- | --- | --- | --- | --- | --- | --- | --- | --- |
| 205 | 17.26±0.96 | 14.74±0.5 | 40.94±1.64 | 7.15±0.83 | 99.06±1.09 | 6.95±0.84 | 3.05±0.11 | 8.57±0.03 | 3.44±0.09 |
| 216 | 13.37±0.09 | 11.94±2.6 | 23.67±0.43 | 7.99±0.37 | 100.53±5.23 | 7.53±0.14 | 3.34±0.18 | 9.4±0.19 | 3.78±0.06 |
| 242 | 12.89±1.32 | 20.64±3.62 | 29.59±1.5 | 6.09±0.77 | 143.75±3.61 | 5.81±0.07 | 5.33±0.79 | 10.46±6.12 | 3.32±0.04 |
| 304 | 18.44±0.9 | 14.86±2.9 | 43.52±9.98 | 7.96±1 | 86.29±2.31 | 6.28±0.82 | 4.29±0.13 | 12.86±0.2 | 3.65±0.08 |
| 313 | 10.35±3.65 | 7.8±4.76 | 17.58±2.27 | 5.38±1 | 65.92±0.08 | 8.96±0.55 | 3.58±0.69 | 8.12±1.1 | 3.54±0.03 |
| 331 | 17.25±0.15 | 17.25±2.97 | 34±1.53 | 7.96±1.47 | 80.56±0.21 | 5.36±0.01 | 6.08±0.07 | 8.37±0.29 | 3.71±0.03 |
| 336 | 13.79±0.33 | 8.9±0.85 | 35.54±4.99 | 6.5±0.5 | 87.89±10.77 | 8.36±0.11 | 3.35±0.46 | 8.55±1.65 | 3.64±0.1 |
| 362 | 17.08±0.21 | 12.64±0.66 | 34.63±4.33 | 8.79±0.45 | 86.87±0.82 | 5.28±0.05 | 3.29±0.31 | 11.25±0.24 | 3.57±0.08 |
| 667 | 18.67±0.61 | 19.95±4.2 | 34.99±5.64 | 8.31±2.69 | 76.67±0.62 | 5.39±0.04 | 5.64±0.2 | 8.83±0.26 | 5.3±0.9 |
| Dahyeon | 10.21±0.15 | 7.34±0.11 | 17.14±1.02 | 7.43±0.33 | 67.73±1.73 | 8.07±0.32 | 3.11±0.01 | 12.99±1.52 | 3.63±0.02 |
| Sunhwa | 13.37±1.62 | 8.87±0.33 | 32.65±2.28 | 8.69±0.69 | 73.24±0.71 | 7.39±0.12 | 3.19±0.24 | 11.47±1.01 | 3.54±0.02 |
| SM1409 | 11.51±0.94 | 5.75±0.17 | 28.7±4.27 | 5.59±0.11 | 48.51±8.48 | 5.81±0.26 | 3.24±0.03 | 9.52±0.58 | 3.81±0.1 |
| **Genotype** | **Genistin** | **Myricetin** | **Resveratrol** | **Daidzein** | **Quercetin** | **Genistein** | **Coumestrol** | **Formononetin** | **Biochanin A** |
| 205 | 30.14±3.12 | 12.01±0.81 | 3.08±0.03 | 3.79±0.33 | 9.2±0.26 | 2.72±0.13 | 4.59±0.24 | 6.16±0.57 | 20.88±1.43 |
| 216 | 28.08±2.76 | 9.28±1.02 | 3.1±0.04 | 4.04±0.1 | 7.98±0.03 | 2.67±0.02 | 6.3±0.17 | 5.06±0.23 | 23.36±0.46 |
| 242 | 25.11±4.12 | 7.18±0.65 | 2.98±0.02 | 3.68±0.44 | 5.91±0.09 | 2.52±0.02 | 5.14±0.35 | 4.28±0.11 | 21.26±0.41 |
| 304 | 31.63±5.45 | 11.61±1.97 | 3.04±0.03 | 4±0.22 | 8.3±0.84 | 2.83±0.08 | 5.37±0.56 | 6.19±1.4 | 22.6±0.01 |
| 313 | 17.31±2 | 7.05±0.16 | 2.95±0.03 | 3.73±0.2 | 5.49±1.23 | 2.72±0.02 | 5.42±0.14 | 4.42±0.98 | 22.42±0.61 |
| 331 | 34.74±3.48 | 12.55±0.29 | 2.99±0.02 | 3.53±0.2 | 6.45±0.85 | 2.93±0.08 | 7.72±1.29 | 7.03±0.21 | 15.37±1.06 |
| 336 | 23.7±3.86 | 9.68±0.15 | 2.96±0.03 | 3.61±0.17 | 6.37±0.02 | 2.9±0.01 | 5.87±0.33 | 5.99±0.41 | 16.63±2.47 |
| 362 | 28.4±2.07 | 10.94±0.53 | 3.04±0.02 | 3.89±0.05 | 11.44±0.16 | 2.63±0.03 | 6.4±0.3 | 7.02±0.86 | 20.63±0.97 |
| 667 | 35.9±1.14 | 13.72±1.1 | 3.09±0.03 | 3.6±0.06 | 6.71±0.09 | 3.15±0.08 | 6.86±0.56 | 7.11±0.45 | 13.95±1.27 |
| Dahyeon | 15.24±0.78 | 10.17±0.15 | 2.91±0.03 | 3.61±0.05 | 6.44±0.05 | 2.85±0.01 | 5.69±0.37 | 3.96±0.05 | 17.06±1.41 |
| Sunhwa | 23.32±3.97 | 11.33±0.69 | 3.02±0.01 | 3.8±0.09 | 6.98±0.45 | 2.94±0.01 | 6.21±0.13 | 5.41±0.94 | 22.11±0.11 |
| SM1409 | 13.13±1.34 | 6.27±0.19 | 2.92±0.01 | 3.68±0.04 | 6.73±0.27 | 2.93±0.21 | 4.99±0.51 | 4.57±0.19 | 19.45±0.3 |

**Supplementary table 4**. Raw Data statistics. (1-1, 1-2 = 205; 2-1, 2-3 = 304; 3-1, 3-2 = 667 ; 4-2, 4-3 = 313 ; 5-1, 5-3 = Dahyeon ; 6-1, 6-3 = Sunhwa )

| Sample ID | Total bases(bp) | Total read | GC(%) | Q20(%) | Q30(%) | Aligned(%) |
| --- | --- | --- | --- | --- | --- | --- |
| 1-1 | 7,212,438,482 | 71,410,282 | 45.1 | 98.5 | 95.5 | 96.76% |
| 1-2 | 7,434,070,256 | 73,604,656 | 44.7 | 98.7 | 95.9 | 97.12% |
| 2-1 | 7,454,074,518 | 73,802,718 | 44.6 | 98.7 | 95.9 | 97.22% |
| 2-3 | 7,279,792,554 | 72,077,154 | 44.8 | 98.5 | 95.5 | 97.19% |
| 3-1 | 6,074,353,312 | 60,142,112 | 44.6 | 98.5 | 95.5 | 96.73% |
| 3-2 | 7,598,738,232 | 75,235,032 | 44.9 | 98.6 | 95.8 | 96.94% |
| 4-2 | 6,047,601,038 | 59,877,238 | 44.8 | 98.5 | 95.4 | 96.70% |
| 4-3 | 6,020,304,576 | 59,606,976 | 44.8 | 98.4 | 95.3 | 96.83% |
| 5-1 | 6,536,525,474 | 64,718,074 | 45.2 | 98.5 | 95.4 | 96.72% |
| 5-3 | 6,277,998,400 | 62,158,400 | 44.9 | 98.5 | 95.5 | 96.95% |
| 6-1 | 6,155,605,186 | 60,946,586 | 44.6 | 98.5 | 95.6 | 97.38% |
| 6-3 | 6,718,033,584 | 66,515,184 | 44.9 | 98.5 | 95.6 | 97.06% |

**Supplementary table 6**. List of 23 Key differentially expressed genes in the biosynthetic pathways of secondary metabolites. N.A., not available.

| Gene name | Gene ID | Log₂ Fold Change | P-value | FDR | EC number | Description |
| --- | --- | --- | --- | --- | --- | --- |
| CCoAOMT_1 | Vradi02g00000724.1 | 5.970 | 1.95.E-10 | 7.76.E-09 | 2.1.1.104 | caffeoyl-CoA O-methyltransferase |
| CCoAOMT_2 | Vradi03g00001121.1 | 5.460 | 1.64.E-03 | 8.02.E-03 | 2.1.1.104 | caffeoyl-CoA O-methyltransferase |
| CYP81E1_1 | Vradi09g00002897.1 | -10.850 | 2.94.E-16 | 8.18.E-14 | 1.14.14.89 | 4'-methoxyisoflavone 2'-hydroxylase |
| CYP81E1_2 | Vradi02g00004162.1 | -5.940 | 7.71.E-10 | 2.57.E-08 | 1.14.14.89 | 4'-methoxyisoflavone 2'-hydroxylase |
| DFR | Vradi07g00001336.1 | -1.240 | 2.11.E-05 | 1.91.E-04 | 1.1.1.219 | dihydroflavonol-4-reductase |
| HCT | Vradi07g00000614.1 | 3.410 | 9.60.E-08 | 1.72.E-06 | 2.3.1.133 | shikimate O-hydroxycinnamoyltransferase |
| AtPCBER1 | Vradi09g00001597.1 | -13.946 | 3.37E-25 | 5.9E-21 | 1.3.1.45 | 2'-hydroxyisoflavone reductase |
| PRX52 | Vradi03g00000817.1 | -6.887 | 1.23E-13 | 1.44E-11 | 1.11.1.7 | peroxidase P7 |
| PRX52 | Vradi05g00000443.1 | -4.574 | 1.93E-06 | 2.36E-05 | 1.11.1.7 | cationic peroxidase 1 |
| PRX52 | Vradi04g00000938.1 | -2.590 | 1.65E-12 | 1.27E-10 | 1.11.1.7 | cationic peroxidase 1 |
| UGT73D1 | Vradi04g00002377.1 | -6.967 | 3.75E-13 | 3.73E-11 | 2.4.1.- | UDP-glycosyltransferase 73D1 |
| ABCG11 | Vradi01g00002787.1 | -3.596 | 1.66E-11 | 9.05E-10 | NA | ATP-binding cassette |
| ATCHS | Vradi09g00003303.1 | -4.841 | 3.44E-19 | 3.04E-16 | 2.3.1.74 | chalcone synthase |
| ATCHS | Vradi09g00003302.1 | -3.241 | 2.13E-15 | 4.56E-13 | 2.3.1.74 | chalcone synthase |
| ATCHS | Vradi02g00001452.1 | -2.500 | 8.75E-15 | 1.51E-12 | 2.3.1.74 | chalcone synthase |
| AtCOMT | Vradi02g00004009.1 | -7.504 | 2.03E-14 | 3.12E-12 | 2.1.1.398 | isoflavone 3'-O-methyltransferase |
| AtCOMT | Vradi07g00000415.1 | -7.376 | 6.94E-22 | 1.53E-18 | 2.1.1.154 | isoliquiritigenin 2'-O-methyltransferase |
| AtCOMT | Vradi07g00000418.1 | -6.892 | 3.43E-12 | 2.36E-10 | 2.1.1.154 | isoliquiritigenin 2'-O-methyltransferase |
| ALDH1A | Vradi03g00000289.1 | -10.746 | 2.18E-07 | 3.51E-06 | 1.2.1.3 | aldehyde dehydrogenase 1A |
| ALDH1A | Vradi02g00003637.1 | -2.965 | 7.30E-10 | 2.46E-08 | 1.2.1.68 | coniferyl-aldehyde dehydrogenase |
| AtPCBER1 | Vradi09g00001600.1 | 3.270 | 2.95E-05 | 0.000256 | 1.3.1.45 | 2'-hydroxyisoflavone reductase |
| LysoPL2 | Vradi04g00001409.1 | -3.982 | 2.83E-20 | 3.98E-17 | 3.1.1.- | caffeoylshikimate esterase |
| SBP | Vradi04g00002565.1 | -5.245 | 3.59E-11 | 1.79E-09 | 1.11.1.7 | peroxidase 3-like |

**Supplementary table 7**. qRT-PCR primer list.

| Gene | Primer | Preimer sequences (5'-3') | Product size(bp) |
| --- | --- | --- | --- |
| CCoAOMT_1 | Forward | CACCACCAGACGAAGGACAA | 107 |
|  | Reverse | GGGCAGTGGAAAGAAGAGAGT |  |
| CCoAOMT_2 | Forward | TGACGCACCGATGATGGATT | 120 |
|  | Reverse | GTAATCCCATCACCCACGGG |  |
| CYP81E1_1 | Forward | CGGATCTCAACCCATCCTCG | 106 |
|  | Reverse | CTTGGACGTGGTGGAGTGAA |  |
| CYP81E1_2 | Forward | GCACGTGGAGATGAGTTCCA | 124 |
|  | Reverse | GAACTCCTTCGCCTTCACCA |  |
| DFR | Forward | TTCAAATACAGCGTGGAAGA | 167 |
|  | Reverse | AAAACAACTTGGCCTTTGAG |  |
| HCT | Forward | AGTTGAGGCCTCTACCGCTA | 108 |
|  | Reverse | GCTTCTGCACCTGTTCTTGC |  |
| EIF5A | Forward | GCACTGACTACCAGCTCATC | 192 |
|  | Reverse | CACAAATCTGCTCCTCTCC |  |
